# Targeted amplification of a sequence of interest in artificial chromosome in mammalian cells

**DOI:** 10.1093/nar/gkz343

**Published:** 2019-05-07

**Authors:** Manami Asoshina, Genki Myo, Natsuko Tada, Koji Tajino, Noriaki Shimizu

**Affiliations:** 1 Graduate School of Biosphere Science, Hiroshima University, Higashi-hiroshima, Hiroshima 739-8521, Japan; 2 Chromocenter Inc., Yonago, Tottori 683-0823, Japan

## Abstract

A plasmid with a replication initiation region (IR) and a matrix attachment region (MAR) initiates gene amplification in mammalian cells at a random chromosomal location. A mouse artificial chromosome (MAC) vector can stably carry a large genomic region. In this study we combined these two technologies with the clustered regularly interspaced short palindromic repeats (CRISPR)/CRISPR-associated nuclease (Cas)9 strategy to achieve targeted amplification of a sequence of interest. We previously showed that the IR/MAR plasmid was amplified up to the extrachromosomal tandem repeat; here we demonstrate that cleavage of these tandem plasmids and MAC by Cas9 facilitates homologous recombination between them. The plasmid array on the MAC could be further extended to form a ladder structure with high gene expression by a breakage–fusion–bridge cycle involving breakage at mouse major satellites. Amplification of genes on the MAC has the advantage that the MAC can be transferred between cells. We visualized the MAC in live cells by amplifying the lactose operator array on the MAC in cells expressing lactose repressor-green fluorescent protein fusion protein. This targeted amplification strategy is in theory be applicable to any sequence at any chromosomal site, and provides a novel tool for animal cell technology.

## INTRODUCTION

**Figure 1.**
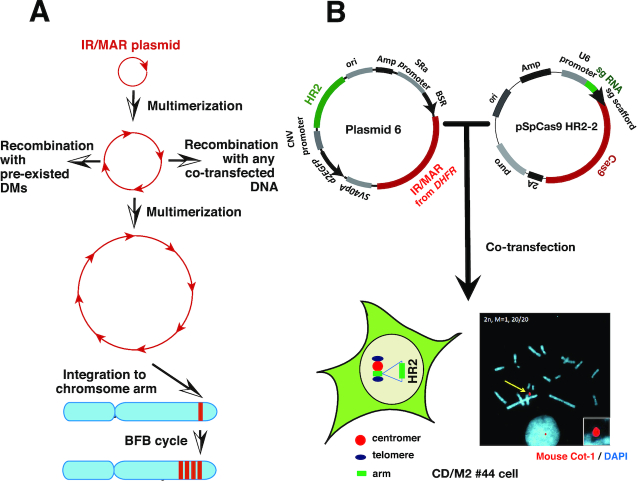
Strategy for amplifying a gene of interest on the MAC. (**A**) Amplification mechanism of IR/MAR plasmid in transfected cells suggested by our previous studies (5,7,10). The plasmid bearing IR/MAR (red arrow) is multimerized as a circular direct repeat in transfected cells that frequently recombines with DMs or any co-transfected DNA and can integrate into random sites in the chromosome arm; the repeat is further elongated by the BFB cycle in the chromosomal context. (**B**) Plasmid 6 has an HR2 sequence homologous to the MAC, an IR/MAR from the *dihydrofolate reductase* (*DHFR*) locus, and blasticidin resistance gene (*BSR*). The pSpCas9 HR2-2 plasmid expresses Cas9 protein and guides RNA specific to the center of the HR2 sequence; it has a puromycin resistance gene (*puro*). The two plasmids were co-transfected into MAC-bearing CHO DG44 cells (CD/M2 #44 cells). The photograph shows a FISH image where the MAC was detected by hybridizing Cot-1 DNA probe (red); chromosomes were counterstained with DAP (blue).

Gene amplification plays a critical role in the malignant transformation of human cells. Amplified genes localize at extrachromosomal double minutes (DMs), episomes, or chromosomal homogeneously staining regions (HSRs) (1–3). Cytogenetically visible DMs and invisible episomes are both autonomously replicating circular molecules without centromeres. We previously found that plasmids with a mammalian replication initiation region (IR) and matrix attachment region (MAR) are spontaneously and efficiently amplified in transfected mammalian cells, generating DMs and/or HSRs (4,5). It has been suggested that IR/MAR plasmids mimic gene amplification in cancer cells, and we previously described the mechanism underlying this process (Figure 1A) (6–8). The IR/MAR plasmid requires replication initiation sequence elements for amplification in cells (9,10). The plasmid is initially amplified at an extrachromosomal site and generates a large circle comprising transfected plasmid sequences arranged as a direct repeat (5). If amplification proceeds extensively, the large circle appears as cytogenetically visible DMs and may be integrated into the chromosome arm, possibly at a double-stand break (11,12). Thus, integration sites tend to be random (8). After chromosomal integration, breakage at or near the amplified plasmid repeat induces a breakage–fusion–bridge (BFB) cycle (13–15) that further amplifies the plasmid to generate a homogeneous or ladder-type HSR (6,8).

We have used the IR/MAR gene amplification strategy in many studies of basic cell biology as well as for recombinant protein production (16–18). Although the increase in gene copy number enhanced protein production, epigenetic repeat-induced gene silencing (RIGS) was frequently observed (19). We isolated a genomic sequence (B-3-31) that alleviates RIGS (20) and also found that *in vitro*-ligated repeat DNA comprising the core IR (G5) (10) can alleviate silencing of the amplified plasmid (21).

The artificial chromosome vector (22) developed by deleting most of the chromosome arm has many advantage over other vector systems. For instance, it can carry extraordinarily large genomic segments (23,24); is highly stable during cell division and is not subjected to selective pressure (25,26); and can be transferred to other cells by microcell-mediated chromosome transfer (MMCT) (22,23,25). In the present study, we combined IR/MAR gene amplification, mouse artificial chromosome (MAC), and clustered regularly interspaced short palindromic repeats (CRISPR)/CRISPR-associated nuclease (Cas)9 technologies to efficiently amplify a sequence of interest on the MAC. We also visualized the MAC in live cells by amplifying a lactose operator (LacO) sequence on the MAC in cells expressing a lactose repressor-green fluorescent protein fusion protein. This method can in theory be used to amplify any sequence at any chromosomal target site.

## MATERIALS AND METHODS

### MAC-bearing Chinese hamster ovary (CHO) DG44 cells

The MAC2 was originally developed by deleting most of the arm of mouse chromosome 11 in chicken DT40 cells and was transferred to hamster CHO cells (22). In this study we transferred the MAC2 described in that report to A9 cells (Supplementary Figure S1). Hereafter we refer MAC2 as simply MAC. The efficiency of IR/MAR gene amplification depends on the cell line; plasmids are efficiently amplified in CHO DG44 but not in CHO K1 cells (17). We therefore transferred the MAC from A9 to CHO DG44 cells by MMCT (22). The origin and culture of CHO DG44 cells has been described elsewhere (17). We obtained 55 clones following MMCT and screened 32 of these by polymerase chain reaction (PCR) using primer sets specific to the MAC sequence to identify MAC-bearing clones (Supplementary Figure S2). These were further examined by fluorescence in situ hybridization (FISH) using a probe for mouse Cot-1 DNA (Supplementary Figure S3). We ultimately isolated three clones (CD/M2 #10, #25, and #44) containing a single MAC that independently segregated during mitosis and was not attached to normal chromosomes. We used clone CD/M2 #44 cells for experiments; 96–100% of cells derived from this clone had a single MAC harboring a hygromycin B resistance gene and a modal number of chromosomes (20 or 21) in CHO DG44 cells. We cultured CD/M2 #44 cells in Ham's F12 medium supplemented with 10% fetal calf serum with or without 150 μM hygromycin B. Culturing the cells for 2 to 3 months in the absence of the drug did not result in loss of the MAC.

### Plasmids

An IR/MAR plasmid bearing a sequence homologous to the MAC was constructed and is referred to as ‘plasmid 6’ (Figure 1B). The pΔBM.d2EGFP vector (27) harboring an IR/MAR from the Chinese hamster *dihydrofolate reductase* (*DHFR*) locus, *blasticidin resistance* (*bsr*) gene expression cassette and intracellular long-lived decay 2 enhanced green fluorescent protein (*d2EGFP*) expression cassette was used as the starting plasmid. We PCR-amplified a 2844 bp sequence in the homologous region (HR)2 sequence (Supplementary Figure S4) located on the MAC (Supplementary Figure S2) using a primer set with a 20-nt HR2-specific sequence linked at the 5′-end to a 15-nt vector cloning site sequence. The HR2 was derived from a genomic sequence proximal to centromere of mouse chromosome 11 and contained 25 759–29 575 bp of BAC clone RP24-239M11 in https://www.ncbi.nlm.nih.gov/nuccore/BX572640). The PCR product was inserted into the *Mlu*I site of pΔBM.d2EGFP using the In-Fusion HD Cloning Kit (Takara Bio, Otsu, Japan). pSpCas9(BB)-2A-Puro (PX459) v.2.0 was obtained from Addgene (Cambridge, MA, USA; cat. no. 62988). The guide RNA sequence in HR2 (Supplementary Figure S4) was designed using CRISPRdirect (https://crispr.dbcls.jp/). Double-stranded oligonucleotides containing HR2-1 (forward, CACCGGCATTACAGTCACAGTTGTC and reverse, CCGTAATGTCAGTGTCAACAGCAAA) and HR2-2 (forward, CACCGGCTGAACAAGAAAGCTAGTA and reverse, CCGACTTGTTCTTTCGATCATCAAA) corresponding to positions 1017 and 1637 in HR2, respectively, were synthesized and inserted into the BbsI/BbsI sites of pSpCas9(BB)-2A-Puro (PX459) v.2.0. The resultant plasmids were named pSpCas9 HR2-1 and pSpCas9 HR2-2, respectively. All plasmids were cloned in *Escherichia coli* DH5α cells. Plasmid DNA was purified using the PureLink HiPure Plasmid Midiprep Kit (Invitrogen, Carlsbad, CA, USA).

### Transfection and selection

**Figure 2.**
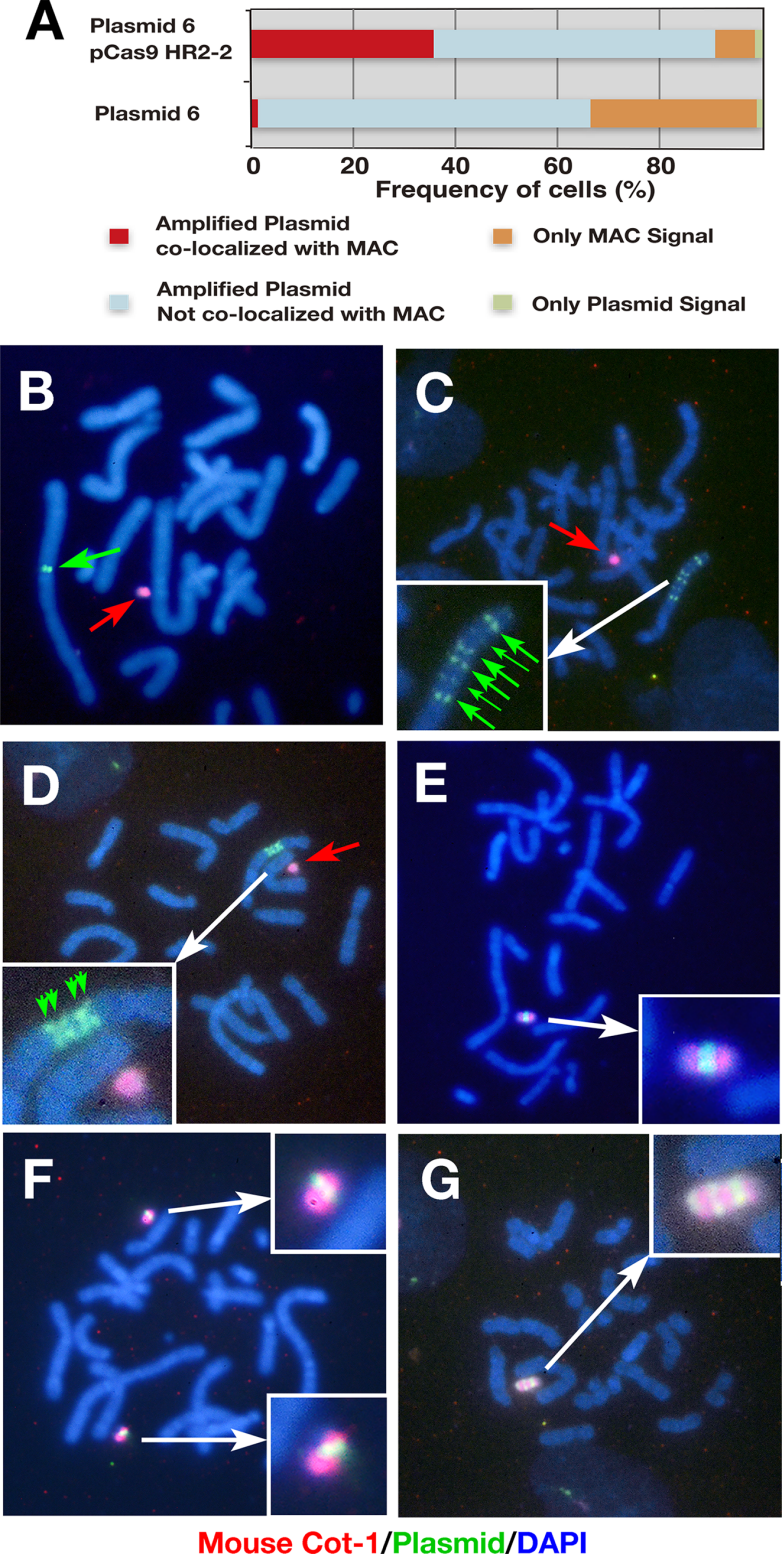
Targeted amplification of the IR/MAR plasmid on the MAC. (**A**) Percentage of cells showing overlapping plasmid and MAC signals. (**B–G**) Representative FISH images of mouse Cot-1 probe specifically hybridizing to the MAC (red punctae and arrows) and hybridized plasmid probe (green punctae and arrows). DNA was counterstained with DAPI (blue). Insets in panels C–G show enlarged images.

**Figure 3.**
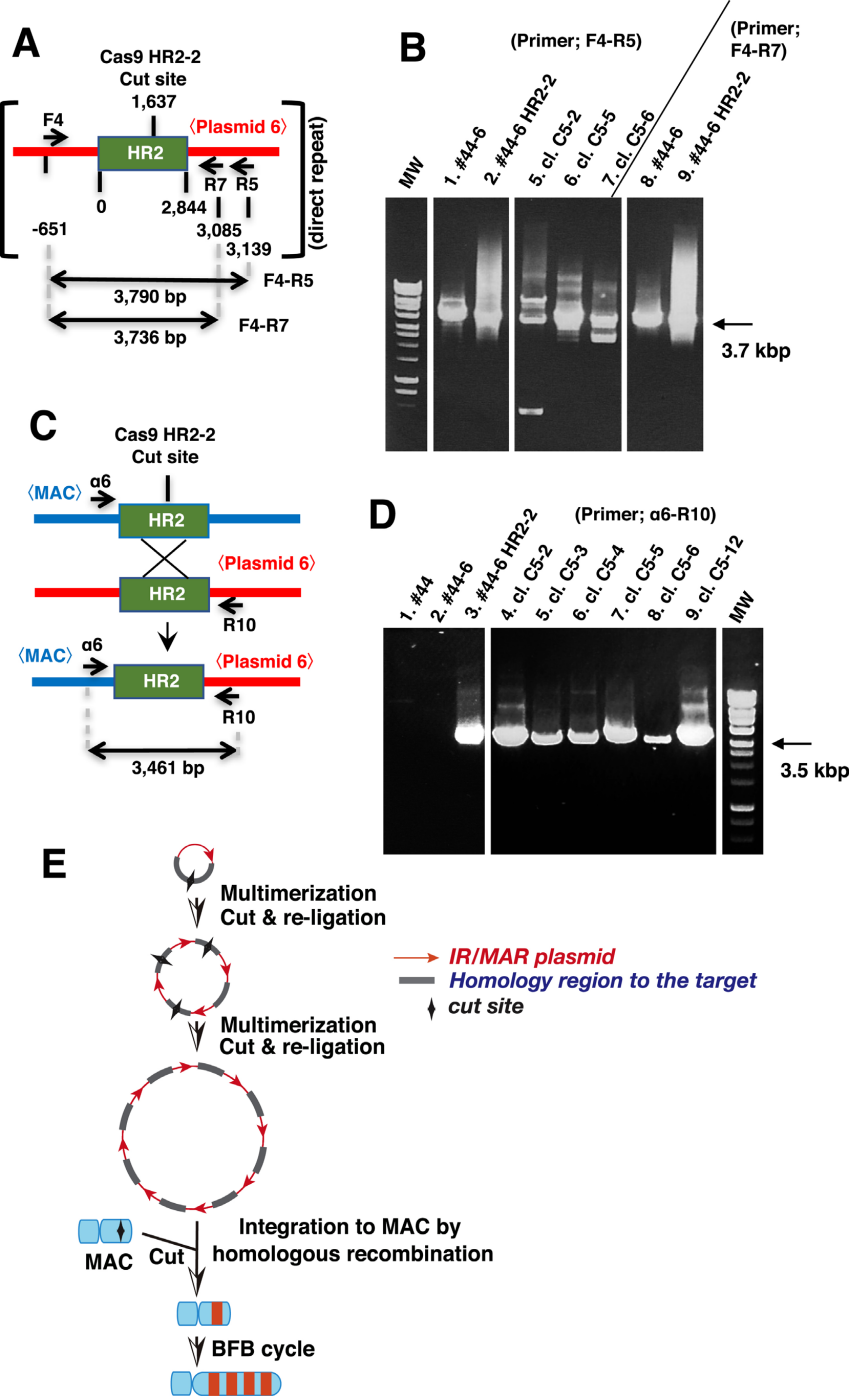
Mechanism of targeted amplification inferred from recombinant structure. (**A–D**) Plasmid–plasmid (A and B) or plasmid–MAC (C and D) recombination was analyzed by PCR using indicated primer sets flanking HR2, followed by agarose gel electrophoresis. Template DNA was isolated from untransfected CDM#44 (#44) cells, CDM#44 cells stably transfected with plasmid 6 (#44-6) or co-transfected with plasmid and pCas9 HR2-2 (#44-6 HR2-2), or clones (cl.) isolated from #44-6 HR2-2. (**E**) Model of the mechanism of targeted amplification inferred from the results of this study combined with the previous model of IR/MAR gene amplification (Figure 1A).

**Figure 4.**
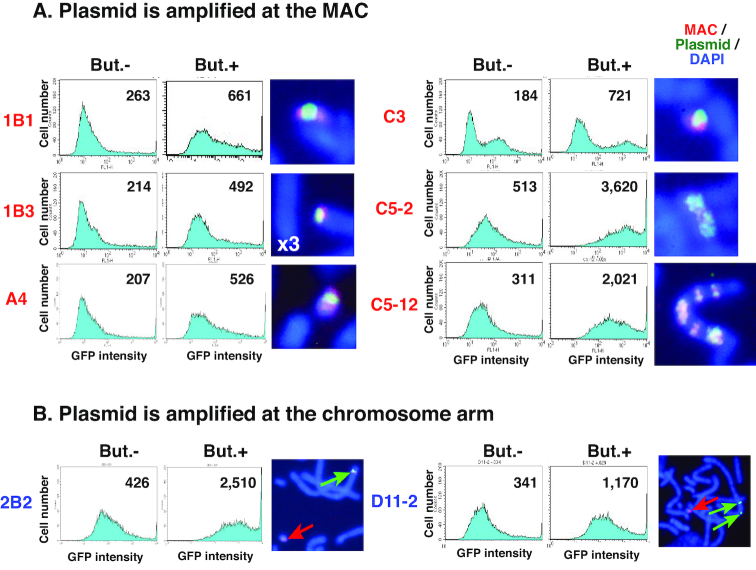
Gene expression from amplified plasmid inside the MAC or normal chromosome. Cell clones were obtained from co-transfection of plasmid 6 and pCas9 HR2-2. For the logarithmically growing cells (But.−) or cells treated with 3 mM sodium butyrate for 3 days (But.+), green fluorescence from the *d2EGFP* gene product was analyzed by flow cytometry. Average fluorescence intensity is shown in each chart. Metaphase chromosome spreads from these clones were analyzed by dual-color FISH to detect the MAC (red) and plasmid (green); representative images are shown. For clone 1B3, each cell had three MACs (× 3) bearing the amplified plasmid.

On the day before transfection, 8 × 10^5^ CD/M2 #44 cells were seeded in a 35-mm plastic Petri dish (Sumitomo Bakelite, Utsunomiya City, Japan). For experiments shown in Figures 2–4, plasmid 6 and pSpCas9 HR2-2 DNA were mixed at a 4:1 (w/w) ratio, and 4 μg DNA were transfected into cells using Lipofectamine 2000 (Invitrogen) according to the manufacturer's protocol. The next day, cells were expanded in a 94-mm Petri dish (Sumitomo Bakelite), and at 2 days after transfection 5 μg/ml blasticidin S (Wako Pure Chemical Industries) and 5 μg/ml puromycin (Nacalai Tesque, Kyoto, Japan) were added to the culture. About 2 weeks after transfection, there were 500–1000 colonies per dish and these were sub-cultured at a 3- to 6-day interval. Metaphase chromosome spreads were prepared about 30 days after transfection according to the standard protocol.

### FISH and flow cytometry analysis

Probe preparation and FISH were performed according to a previously published protocol (28). Mouse Cot-1 DNA was purchased from Invitrogen. Mouse major satellite DNA was PCR-amplified according to a published protocol (29). The DNA was labeled with biotin using the BioPrime DNA Labeling System (Thermo Fisher Scientific, Waltham, MA, USA). Digoxigenin (DIG)-labeled plasmid probe was prepared using BioPrime DNA Labeling System and DIG-DNA labeling mixture (Sigma-Aldrich, St. Louis, MO, USA). Alexa Fluor 594-conjugated streptavidin (Invitrogen) and fluorescein-conjugated sheep anti-DIG antibody Fab fragments (Roche Diagnostics, Basel, Switzerland) were used to detect the hybridized probe. The ECLIPSE TE2000-U system (Nikon, Tokyo, Japan) equipped with a 60 × objective lens (Plan Apo VC 60 × /1.40 oil; Nikon) was used for imaging. For flow cytometry analysis of *d2EGFP* expression, cells were resuspended in phosphate-buffered saline (PBS) and sorted on a FACS Calibur instrument (BD Biosciences, Franklin Lakes, NJ, USA).

### PCR

Total DNA was extracted from cells according to a standard protocol by sodium dodecyl sulfate lysis and phenol extraction/ethanol precipitation. Primers for amplifying the plasmid region flanking HR2 had the following sequences: F4, TGGATACAACGTATGCAATGG; R5, TCTACCTGCCTGGACAGCAT; and R7, GCGAGAAGAATCATAATGGG. Primers for amplifying the recombination junction between the plasmid and MAC had the following sequences: α6, GCTGGTAGGCAAGACCAATTAATTACTGGC and R10, GAGATGGCGGACGCGATGGATATGTTCTGC. Gflex DNA polymerase (Takara Bio) was used for amplification.

### Visualization of the MAC

**Figure 5.**
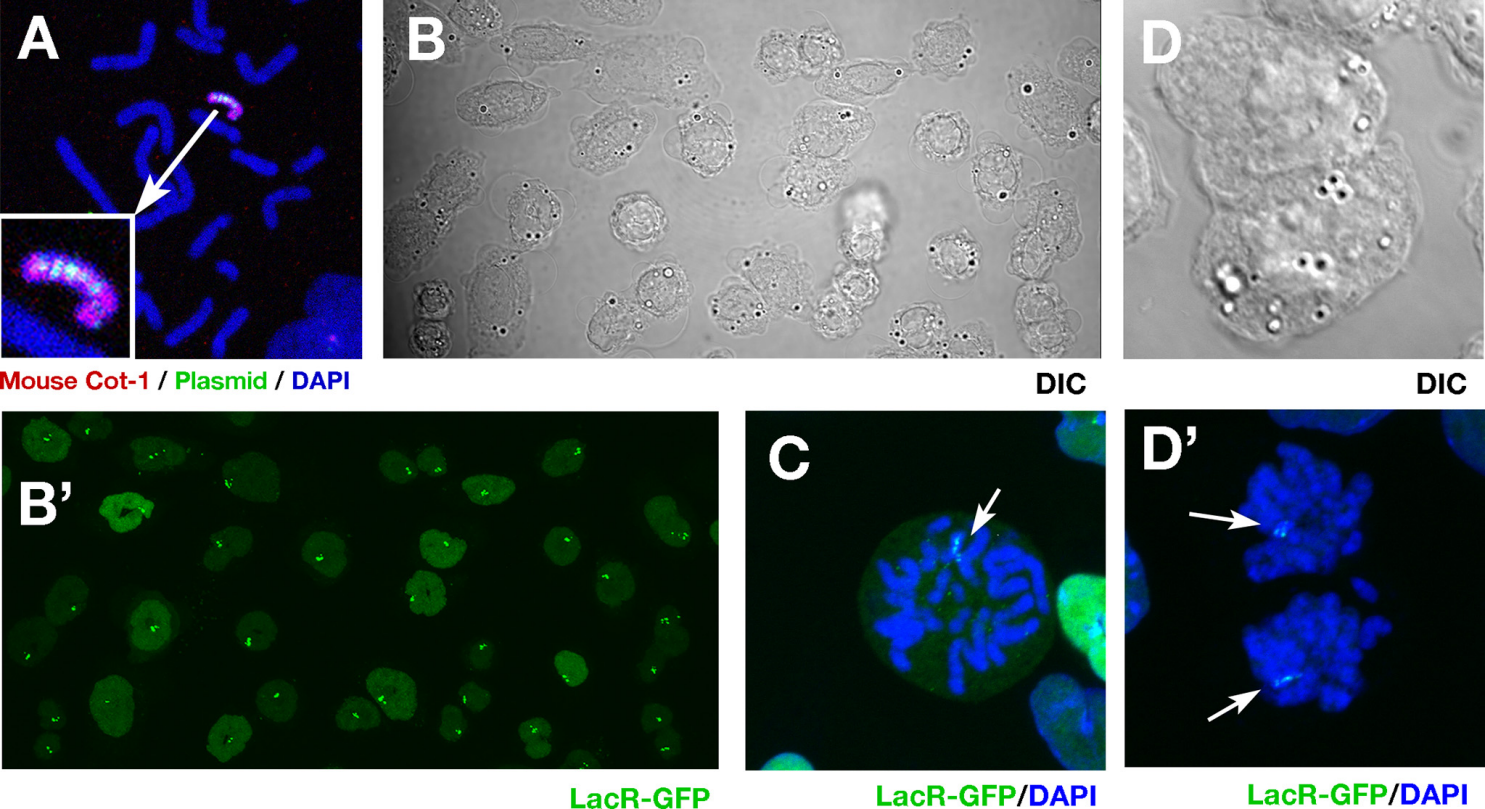
Visualization of MAC in live cells. (**A**) Metaphase chromosome spread from clone E5 cells analyzed by hybridizing plasmid (green) and mouse Cot-1 (red) probes. (**B–D**) Clone E5 cells grown in a glass-bottomed dish were fixed in paraformaldehyde and stained without (B) or with (C and D) DAPI. Differential interference contrast (DIC; gray), DAPI (blue) and LacR-GFP (green) images of logarithmically growing cells (B) or cells in prometaphase (C) or telophase (D) were obtained on a confocal microscope.

The lactose repressor (LacR)-GFP fusion protein with nuclear localization signal was expressed in CD/M2 #44 cells as previously described (6). Several clones with weak and uniform green fluorescence in the nucleus were obtained; we used clone 2A1 in this study. Cells derived from this clone were transfected with a DNA mixture of plasmid 6 (2.4 μg), HR2-2 (0.6 μg) and pSV2-dhfr 8.32 (1.0 μg). The last plasmid has 10.1 kbp of the *LacO* repeat sequence and was used in our previous study (6). The transfected cells were selected with 5 μg/ml blasticidin and 7 μg/ml puromycin. A total of 28 independent clones were obtained by the limiting dilution method; these were individually seeded in collagen-coated 35-mm glass-bottomed dish (MatTek, Ashland, MA, USA) and examined under an epifluorescence microscope (ECLIPSE TE2000-U; Nikon, Tokyo, Japan) equipped with a 100 × (Plan Fluor 1.30 oil; Nikon) objective lens. Clones with GFP punctae in the uniform background GFP fluorescence of the nucleus were selected and cultured. We prepared metaphase chromosome spreads of these cells and performed dual-color FISH using biotin-labeled Cot-1 and DIG-labeled plasmid probes. We selected clones with overlapping Cot-1 and plasmid signals. For images appearing in Figure 5B–D, cells cultured in a 35-mm glass bottomed dish were fixed with 2% paraformaldehyde in PBS for 10 min and stained with or without DAPI. Images were acquired on an LSM700 laser scanning confocal microscope (Zeiss, Oberkochen, Germany) and 15–20 z-series images acquired at a 0.8- to 1- μm interval were merged (Figure 5B’, C and D’) in Adobe Photoshop CC (Adobe Systems, San Jose, CA, USA). A differential interference contrast (DIC) image of a single representative section is also shown in Figure 5B and D.

## RESULTS AND DISCUSSION

### Amplification of IR/MAR plasmid in the MAC vector

We used CHO DG44 cells for this study since they support efficient IR/MAR gene amplification (17). We transferred the MAC from A9 cells to CHO DG44 cells and screened 32 clones by PCR followed by FISH (Supplementary Figures S2 and 3); CD/M2 #44 cells were used in subsequent experiments. Cells derived from this clone had a modal number of chromosomes (20 or 21) in CHO DG44 cells and a single MAC that was stably segregated during cell growth. We initially hypothesized that there would be frequent recombination between the IR/MAR plasmid and the MAC, since the plasmid is known to recombine with co-transfected molecules or pre-existing DMs in the same cell. Furthermore, the IR/MAR plasmid is amplified to a tandem repeat in an extrachromosomal context (5,9,10), which may facilitate homologous recombination (HR) with the target. We therefore inserted a 2844-bp HR2 sequence homologous to the MAC into the IR/MAR-bearing pΔBM.d2EGFP plasmid to generate plasmid 6 (Figure 1B), which was transfected into CDM #44 cells. Stable transfectants were selected with blasticidin for around 30 days before they were examined by dual-color FISH using a plasmid probe and the mouse Cot-1 DNA probe to detect the MAC. Although the plasmid was amplified in the cells, there was little overlap between its signal and that of the MAC (Figure 2A and data not shown), suggesting that recombination between the IR/MAR plasmid and MAC occurred too infrequently for practical use.

We next used the CRISPR/Cas9 system to improve specificity for the MAC. We constructed a pSpCas9 HR2-2 plasmid expressing Cas9 nuclease and a guide RNA specific to HR2 in the IR/MAR-bearing plasmid 6 and in the MAC. We then co-transfected pSpCas9 HR2-2 and plasmid 6 into CDM #44 cells and selected stable transfectants with puromycin and blasticidin for about 30 days before FISH analysis. A high percentage (nearly 40%) of cells showed co-localization of the plasmid and MAC signals (Figure 2A). The pSpCas9 HR2-1 plasmid—which targets a different site in HR2—showed a similar result to pSpCas9 HR2-2 (data not shown).

In some metaphase cells, the plasmid (green) was amplified at the chromosome arm independently from the MAC (red; Figure 2B). In another cell, the chromosomal plasmid repeat was further multiplied and appeared as a ladder (Figure 2C and D) in which the symmetrical arrangement of the plasmid signal along the chromosome arm was consistent with a BFB cycle (8,13–15). Importantly, nearly 40% of cells (Figure 2A) showed amplification of the plasmid at the MAC (Figure 2E–G). In some cells, two MACs with amplified plasmid appeared in single cell (Figure 2F). Interestingly, there were cells with a long MAC where the plasmid signal appeared as a ladder (Figure 2G); such structures appeared in 10%-20% of MACs with amplified plasmid. This suggests that the BFB cycle extended the plasmid array in the artificial chromosome context as in the normal chromosomal context.

### Analysis of inter-/intra-plasmid and plasmid-MAC recombination

We isolated total DNA from the above cells or their clones and analyzed the recombination junction by PCR. We first examined the inter- and/or intra-plasmid junction by PCR-amplifying the plasmid sequence surrounding HR2 (Figure 3A) using two primer sets (F4/R5 and F4/R7). When DNA from CD/M2 #44 cells transfected with plasmid 6 alone was used as a template, a sharp band was obtained with a size corresponding to the original sequence with no alterations (∼3.7 kbp; Figure 3B, lanes 1 and 8). On the other hand, co-transfection of plasmid 6 and pCas9 HR2-2 yielded a smear at around 3.7 kbp (Figure 3B, lanes 2 and 9). This suggests that large insertions/deletions at the cutting site were frequent. Furthermore, analysis of randomly selected clones from these cell populations revealed several distinct bands reflecting large insertions/deletions at the cutting site. It is well known that insertions/deletions are common during DNA repair by non-homologous end-joining (NHEJ). Furthermore, since IR/MAR plasmid is multimerized to a direct repeat in cells (5), cutting at multiple sites in the plasmid repeat and re-ligation by NHEJ would produce a heterogeneous pool of PCR products with sequences that cannot be re-cut by the same Cas9/guide RNA.

We next amplified the recombination junction between the IR/MAR plasmid and MAC (Figure 3C). PCR amplification yielded a sharp, single band whose size was predicted from HR between HR2 in the plasmid and in the MAC (Figure 3D). The same result was obtained for stable transformants from the co-transfection of plasmid 6 and pCas9 HR2-2 and for several randomly selected clones. There were no bands for cells before transfection or transfected with plasmid 6 alone, which served as controls. Thus, cutting at HR2 on the MAC facilitates HR with the array of plasmids bearing HR2, indicating that inter-/intra-plasmid recombination is mediated by NHEJ and that HR occurs between the plasmid and MAC.

Based on these results and those of our previous work, we propose the following model (Figure 3E). After transfection, the IR/MAR plasmid is episomally maintained in the cell and amplified to a tandem repeat. Cas9 cleaves HR2 in the plasmid and re-ligation produces uncleavable sequences. Cas9 also cleaves HR2 on the MAC, which facilitates HR between multiple plasmid copies. The plasmid is further amplified in the MAC through the BFB cycle, as discussed below.

### Gene expression from the plasmid array on the MAC

We evaluated the expression of d2EGFP expressed by plasmid 6 by flow cytometry of clonal cells at about 3 months after the initial plasmid transfection. Representative results from eight clonal cells are shown in Figure 4. The FISH analysis revealed that the plasmid was amplified at a single locus on the MAC in four of the clones (1B1, 1B3, A4 and C3). In clone 1B3, three copies of this MAC were detected per cell, suggesting a non-disjunction event during clone isolation. In clone C5-2, the plasmid was amplified as a fine ladder structure on the MAC; in clone C5-12, a structure resembling C5-2 was integrated into the chromosome arm and generated a ladder structure; and in clones 2B2 and D11-2, the plasmid was amplified at the normal chromosomal arm independent of the MAC. The FISH analysis also revealed low intra-clonal heterogeneity of the amplified structure, suggesting that the amplified structure was stable until clone isolation. Flow cytometry analysis of cells in logarithmic growth phase and of those treated with butyrate—which inhibits histone deacetylase activity and thereby partly alleviates epigenetic silencing—showed that gene expression from the amplified plasmid was similar or slightly lower in the context of the MAC as compared to the chromosome arm. In the MAC, centromeric or telomeric heterochromatin may influence amplified sequences located close together. The FISH analysis confirmed the clonality of the cells despite their broad fluorescence profiles, which might be caused by that the amplified transgene was subject to epigenetic chromatin silencing as we previously reported (19) and that gene expression may fluctuate during cell cycle progression (30) or during changes in cells’ physiological state. Interestingly, a higher expression was observed for clones in which the plasmid array in the MAC was further amplified to a ladder-type structure (C5-2 and C5-12), which is typically associated with higher gene expression in the chromosomal context (17).

We previously showed that IR/MAR amplification is dependent on the cell line that is used, with more efficient amplification in CHO DG44 than in CHO K1 cells. Our current results show that high expression was achieved for a plasmid on the MAC, especially as a ladder structure. The MAC was transferrable to another cell line (e.g. CHO K1 cells) by MMCT. This provides a useful strategy for expressing large quantities of valuable proteins such as biopharmaceuticals.

### Visualization of the MAC in live cells

In order to test whether the developed strategy can be applied to any sequence, we amplified the *LacO* sequence on the MAC and visualized it by binding to LacR-GFP in live cells. We established CD/M2 #44 cells that express LacR-GFP with nuclear localization signal and clonally propagated those exhibiting weak green fluorescence at the nucleus. The cells were co-transfected with plasmid 6, pCas9 HR2-2 and pSV2-dhfr.8.32 with a 10-kbp *LacO* repeat based on our previous finding that any sequence co-transfected with the IR/MAR plasmid is co-amplified (5). We selected stable transformants with blasticidin and puromycin and observed several clones with green punctae among uniformly distributed GFP throughout the nucleus under the fluorescence microscope. Metaphase chromosome spreads of cells derived from these clones were analyzed by FISH using the plasmid and mouse Cot-1 probes. More than 95% of cells from clone E5 had the amplified plasmid sequence in the MAC, which was visible as a long ladder-type HSR (Figure 5A); each cell also had a bright nuclear GFP puncta. We were able to trace very bright MACs at interphase (Figure 5B) or during mitosis (Figure 5C and D) in clone E5. The MAC detected by FISH and GFP punctae in the nucleus in live cells indicated that there was one copy of the MAC in over 90% of cells, although 5–10% of cells had two copies. Both the MAC and nuclear GFP signal were highly stable over long-term culture, and were observed in > 90% of cells more than 3 months later without drug selection.

### BFB cycle in the MAC

**Figure 6.**
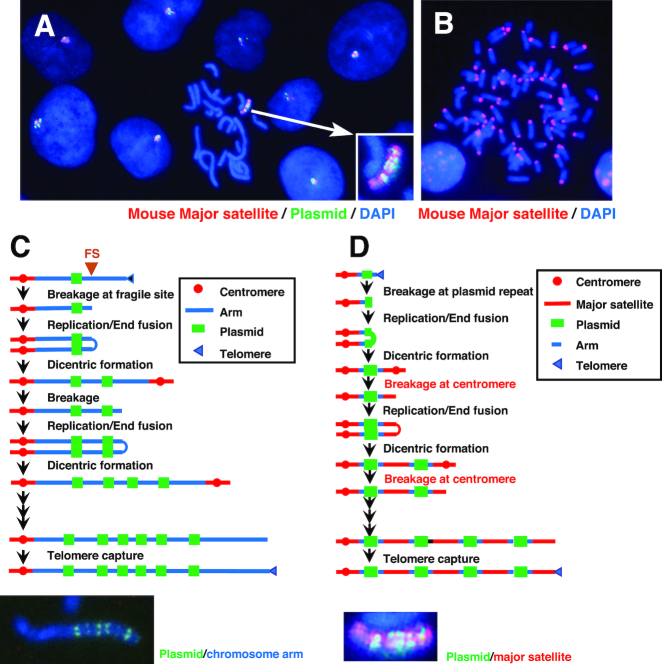
BFB cycle on the MAC. (**A**and**B**) Metaphase chromosome spreads of clone E5 cells (A) or mouse embryonic fibroblasts (B) were hybridized with mouse major satellite probe (red); the plasmid probe (green) was simultaneously hybridized in (A) The BFB cycle in the normal chromosome arm (**C**) and the BFB cycle in the MAC (**D**) are depicted. Both cases involve dicentric chromosome formation; however, they differ in the site of dicentric breakage—the chromosome arm (C) and major satellite (D), respectively. The photograph that represents the resultant ladder structure is shown under each cartoon.

We detected the MAC by FISH using the probe prepared from mouse Cot-1 DNA. As mouse Cot-1 DNA contains highly repetitive sequences in the mouse genome, we also hybridized a probe prepared from a mouse major satellite sequence that constitutes the highly repetitive centromere DNA and is enriched in Cot-1 DNA. We found that it also hybridized along the entire length of the MAC with ladder-type plasmid amplification in the clone E5 (Figure 6A). As a control, the same mouse major satellite probe was hybridized to the centromeric region of acrocentric chromosomes in mouse embryonic fibroblasts (Figure 6B). As described above, the MAC in clone E5 was very stable, suggesting that it has only one active kinetochore. From these results, we propose that the ladder-type MAC is generated by a variation of the normal BFB cycle (8,14,15) (Figure 6C) in which amplification of the transfected plasmid is initiated by a breakage at the chromosomal fragile sites near the plasmid. Replication and end-fusion generate a dicentric chromosome, which generates a second breakage near the plasmid. On the contrary, the BFB cycle in the MAC involves breakage at the major satellite after dicentric chromosome formation that inhibits centromere activation (i.e. kinetochore formation), thereby generating a stable MAC with the entire major satellite and ladder-type arrangement of amplified plasmid. This may be related to the well-known phenomenon that one of the two centromeres in dicentric chromosomes is frequently inactivated by genetic or epigenetic mechanisms (31,32).

### Implications of this study

We developed a method to amplify any sequence on a MAC by combining IR/MAR and a sequence homologous to the target that is cleaved by CRISPR/Cas9. The amplified sequence on the MAC can be further extended to a ladder-type HSR—which increases transgene expression—by a unique form of the BFB cycle involving breakage at the centromeric sequence. The MAC is transferrable to other cells (33) in which amplification is less efficient. Namely, we may amplify a sequence of interest at the MAC in a cell line where the amplification is efficient; then we may transfer the MAC with amplified sequence to another cell line where the amplification is less efficient. Furthermore, the targeted amplification can be applied to any chromosomal region using a sequence homologous to the target chromosome site. Thus, this method provides a novel and useful technology that can be applied to investigate basic cell biology or used for high expression of recombinant proteins in animal cells.

## Supplementary Material

gkz343_Supplemental_File
