# Supplementary material for: Targeted amplification of a sequence of interest in artificial chromosome in mammalian cells
Source: Nucleic Acids Res. 2019 May 7;47(11):5998–6006. doi: 10.1093/nar/gkz343 (PMC6582328; doi:10.1093/nar/gkz343)
Supplement: gkz343_Supplemental_File [file gkz343_supplemental_file.pdf]

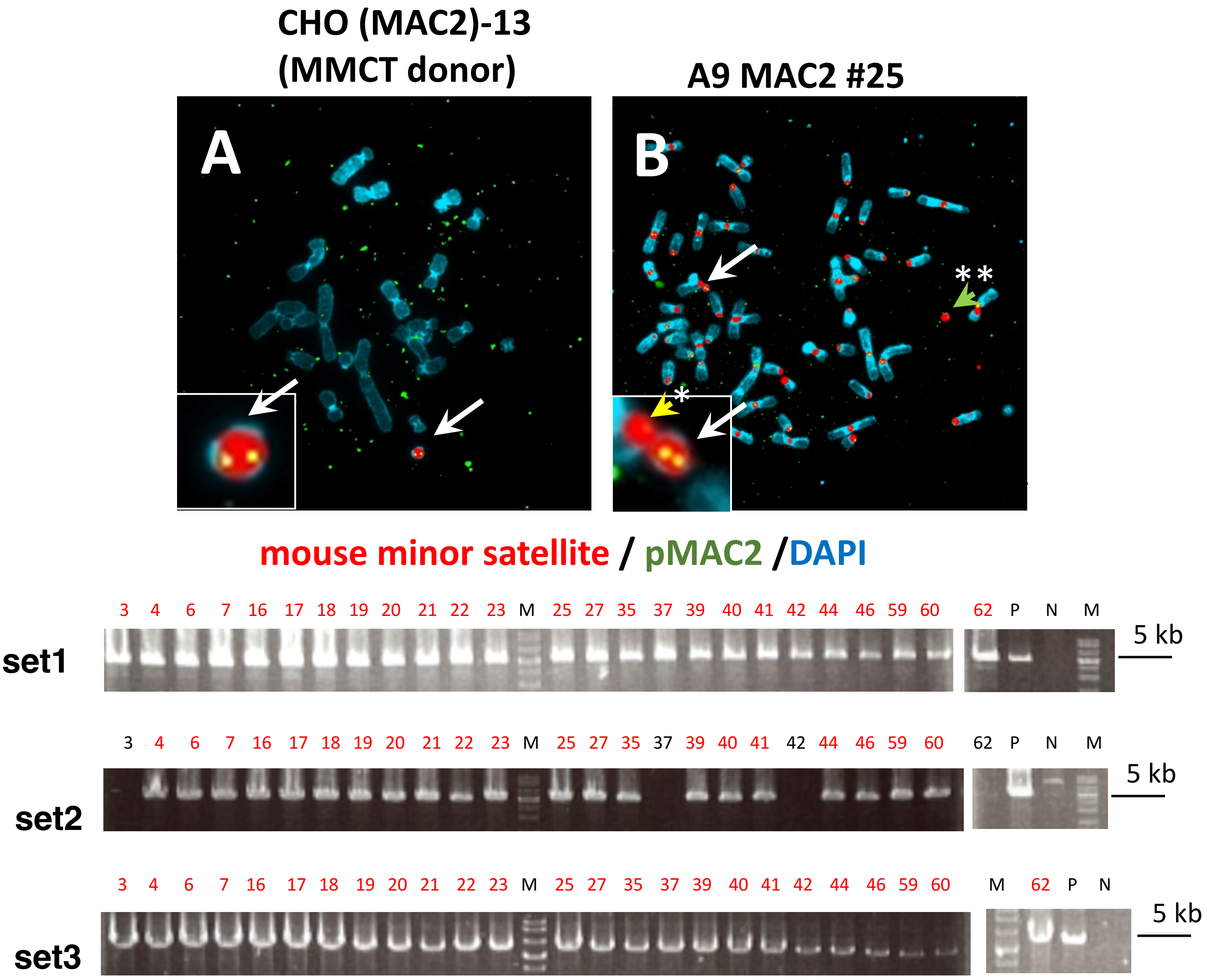

Positive control: CHO (MAC2)-13, Negative control:A9
M: marker, red: positive clone

Supplementary Figure S1. Transfer of MAC from CHO (MAC2)-13 cells to mouse A9 cells by MMCT. **A, B.** The metaphase spread was hybridized with mouse minor satellite (red) and pMAC2 (green) probes. pMAC2 contained 5'HPRT-loxP-Hyg that was inserted into MAC2 (Supplementary Fig. S2A). Since mouse minor as well as major satellites are a centromeric sequence, the MAC hybridized to all centromeres of acrocentric mouse chromosomes in A9 cells (B). The MAC2 contained both a minor satellite and sequence derived from pMAC2, and is visible as a red circle with a pair of green (yellow) dots (white arrow in panels A and B). The yellow arrowhead with an asterisk in B indicates a centromere of an acrocentric mouse chromosome. The green arrowhead with two asterisks indicates a structure commonly seen in A9 cells. DNA was counterstained with DAPI (blue). C. PCR screening of cells bearing the MAC. Total DNA from each clone were PCR amplified using primer set appearing in Supplementary Figure S2A.

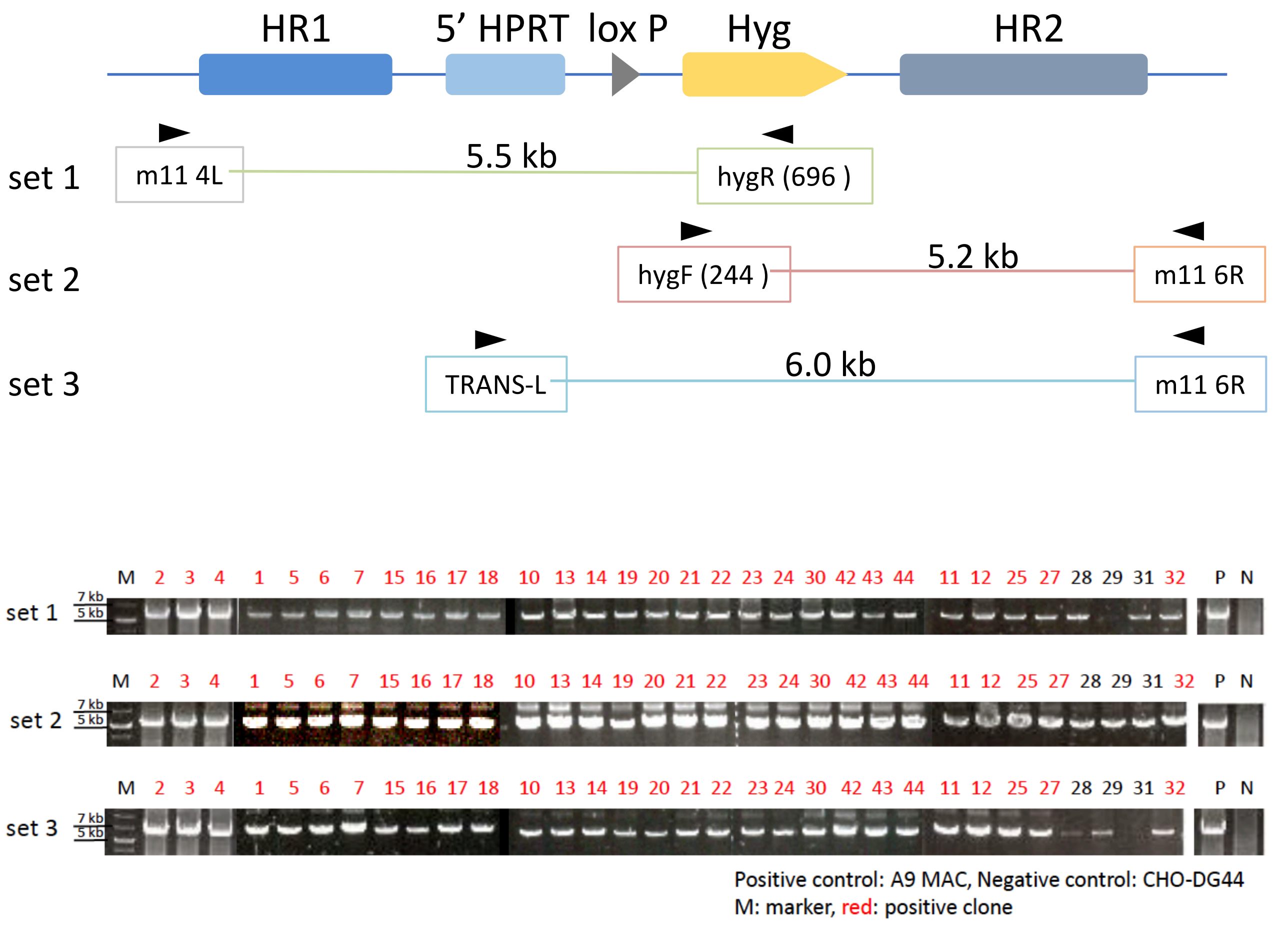

Supplementary Figure S2. Structure of MAC and screen for MAC-bearing clones. **A.** Structure of a portion of the MAC and position of PCR primer sets for detecting the MAC-bearing clone. **B.** PCR screening of cells bearing the MAC.

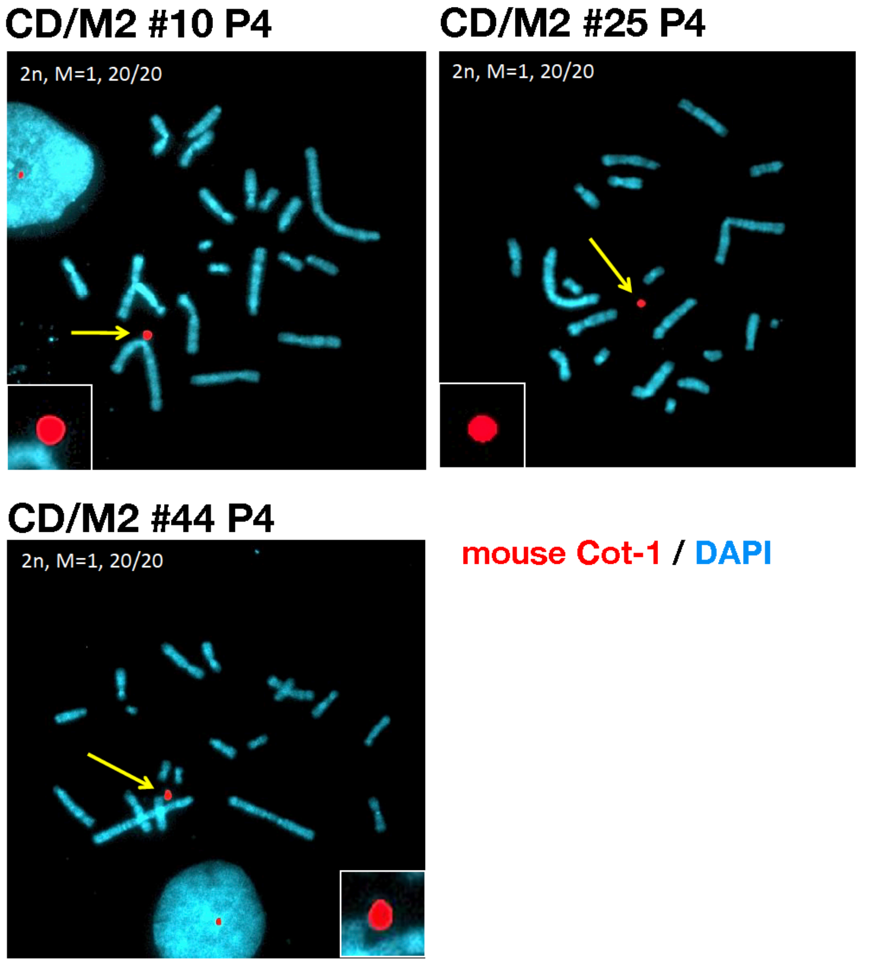

Supplementary Figure S3. Representative FISH images of cells bearing the MAC. Metaphase chromosome spreads were prepared from indicated clones. The MAC (yellow arrow) was detected by hybridizing Cot-1 probe (red), and the chromosome was counterstained with DAPI (blue).

Supplementary Figure S4. Sequence containing HR2. Blue underlined sequence: PCR primer sequence for amplifying the 2844-bp HR2 sequence that was inserted into the *Mlu*I site of pΔBMd2EGFP to obtain Plasmid 6. Red underlined sequence: sequence recognized and cut by Cas9 HR2-1. Pink hatched sequence: sequence recognized and cut by Cas9 HR2-2.

GATCTGCTTTGCAGGAGCACTGGGCTCCCTCTAGTCCTCTCGGTTGTGGCTGCCA
AAGGATCTTTCCACACAGGAGCATGAGGGTCTTTAGCGGAGCCCCAGGATGTCTA
GGTGAGGGGCACTGTGGGGCTGACCTTATAAGACCTGAATGATGTGTTCCTGCCT
GTCCATGGGTGCAGAGGAGAGGTTCTGGGTCTCTCAGGACCTAGGCCCCTTTTCT
GAGTATGGGTGGGTGTGATTTGACCCCAAAGTCACTTTAAGGGGTGTTTTAAATT
TTTGTACAAGCCAGAGCCTCCCACCCCTAACTCCTGGCTGTGTACTACCACACCC
TCTAGCTGCGCCCCGACCCGTCACAGGGCTCTCTCACTGTGCACAGGGTGACGTG
CCTGCTGGGCAGAGACTGTGGATCCACTGTCTTCCTAGAGCAGGCCACATGGCCT
TCATAACTGCTTTCTGCTCCCAAATTTCACCTATCTGCATGCTGGAAAAGTAAAAT
CATTTATATATTGCTGGATGTGTTGTCCTGGGCTCTGTCAGTGTCTTCTGAAGACA
ATGACTAATGATGTATGTAGACCTTTGTAGGCTCTAAGAAGCTGGCTGAATCCTTT
TGCTGGTAGGCAAGACCAATTAATTACTGGCTTGTCTGGTAGAGTCTGGAGTGAA
AGGGCAGGTTGAGGCCTTGCCCTGCAGAAGTAGGGGACATGCTGCTATTGGTCTA
GGTCTGTGTTGGAAGCAGAGGGTATGGAGGTCAGTACCTAGAAAACAGGTATGC
AGGCCATGGGGAACCCAGTGGTAGGGACAAGACCAGAGTTCATGTCGATCTTGG
GAATGCCATGAATTTCCCCTGCAGAAGAAATCACTTAACTTTTCAACCCTATCAGT
AGTACCGTATTTTGTATGAAAAGTAGGAGACTTCTGAACAGTAATTCATTTCATTG
CAAGACATTTTGAAATTAAAAAACAAAACAAAACAAAAACTACCATAGCTTTGAAT
TTTGTTGTTGAGGGTGTCCCAGGCCTTGTGAAGGATAGCACCTATCCCCCAGGGA
CGGGCCACTAGCACAGTGCCCGTCTCAGGGCCCGAACACGAGCAATGCATGTAC
CAACAGGCTGACGCTGGGCCTCAAGCTCCTTAGCCAGCTGCTGGACCTGTAGTTC
CACCTGGGGACATGGGCGAGAGGGAGGGACAAGGCAAGCTGAAAGGAAGGACA
GCCAGGGGCTGGCCCTGGGCTGACCTGGTCTTACCTCCTCCAGTTCTGTTTTCAC
CTGCTCTTCTAGGTGAAGGTCCTCGGATTTCGGCTCCTCCTGGCTTAGCTCCAGC
CACCTACGCAGGCTGTTGGCTTGTCGCTGGCATGACCTGGAGATGGGGACACCTA
TCAGGCTGGGTTCTTGGCACAGCCCCAAGTGAGCCCTTTGAAGAGATATATTGGC
TAATAAAGTGCTTCGAGAGAATGGCCAACAATCTTCTGTCTTCCCCAGCAAACAG
CAACTCATGTGAGCCGTGTGAAAACTTCTCTTCCTGTCCCTTTCTAGAGGGACCA

GTCTTTTTCACTGAGGTCTCTGAAAGCTGCTGTATGTCAACTGTCCCCAGAACACT
GGGTATAAGTAGGACCCAGGCCCACGCTGTCTGTCCCTTGTTAGCATTACAGTCA
CAGTTGTCAGGTAGGTAACAGAACCCAGGAGAGCCTTGCTTGGCATTCCATAGCC
TCCAAATGGAAAGCAGGACTTCCAGCCCTATGTAAGGCCAACTTGGCCCTTCCTG
GAGTGGATAAAGGTGCCAGAGGACACTTACCCCACCCCCTGGAGTTAGACCACAC
AGAGGAGTCCCAGGCCCTGCTGCAGGAAATGGAACCAACCACCTTTCCAAGCCAC
AGCTATTCCCATAGACAGGTCCCAGGGCACCTGGTAGGCCTCACCAAAGGTTCTG
CTTGGTGGTTTGGTAGTGTTGTAACTGCTGCTGGATCCCCTCCAACTCAGCCTCA
AGCTCTGTGTGGCCTGCAGCAACAGGAAGATAAGCTTGACCTTGCCCACCATCCC
TTTAGGACCCAGGAGACTGGTAGGACTTTGGTAGAGGGTGATTCCAGCACCCTGA
CTTACTCACCTGTAGCCTCTGAGCCATAAACAGGCTCAGTCCGGGTGCTTATGGA
ATCTCCAGGGAGGAGTTGGGGATCAAGGTCCCTGGGTAAGGACGGGCACCCCCA
AGGCTGAGGCCCAGGGATGCTGGGCTTCGCTGACACCTGTAGAGGAGCAAAGGG
TGGATGCTGAACAAGAAAGCTAGTAGGGACCCTGGAAAGTGAGTCCTGTCCTGAT
TCATTGCCCACCATGCCTTGCAGTGCTGAGGGTGACTGCCCTGCCTGTTGAGCCT
CAGGGAAGACTGTTACAGGGCAGCACTCCTGTCCTACCACTCCTGCACCCCCAAG
GTTTCTCTTCTGTGTGCCACTATTTTTAAAAATACTAATTTACTGAGACAGAGTC
TTGCTATGAAGCCCAGGCTCATTTTGAGCTCTCTATCCTCCTGCCTCTATTGCCTA
AGTGCTGAGATTGGGTGTATGTCGCTTCTGTTACCAGCCACGCTCCACCAGAAGC
TACCTCCTTCCTGTCTCCACCTCTCCCCAGCCAGCACAAACTTTTAGGAACTGCTG
ACTGGCTGTTTTCAGGAGTACCCCTGACTACCTCTGCCCAGGACTCCGTGTCCCC
TTGTTCTTCGAGGTGAAACTTTGCTGTGCAGTGTAGATACTCATAGCCTTGAAGG
CTGACTGAGTGGGGACTGACTGCCTCACCAGACACTGGACCCTACCCCAGATTCC
ATCTTTTAGTAAAAGAATTCTTTCTTAGTGACCCTGGATTCCTCTTCCTCCCTCAC
CATAACTAACATAGCACGTCCTACCAAAGACAGTCCCCAACAGACTGTCACTCAC
ACACACACACACACACACACACACACAACACACACACACCAGTGAGAAGCTACAG
CCAGAGCACTAGCATGTCTGTCTGATCACACCACAGCTCATGGCTGCCCTAGCAC
TTAGTGCATGAGTCCAGGCCAGATGTGGTACCCTACAACAGGCCCTCAATTTCTT
CCTCTTCCCACTCCAAGCTCCCACCTGTCCCCCAAGACTAGAGGCCCTGCTGGGA
GCCCGCCTATAAGGTTCTTCTGGTTGTCCAGACATCAGTCATTGCTCAAGGGCTG
GGTTGTTAGAAGACTAGCCACTGCTGTGTTTTAGGTCTGGTGTGCAGGGCACCCA
AGCTTGTCAAGTGACCAAGGTATTCTAGAAAGAAGAGTAGTAAGGGTTGGAGAAT
GAGCACCAAAGGAGAGTGAGTGGCAGCACAGTGGAGAACTCACCCTAGCAGTGC
CTCCTGCCGCATGGGGCATGATGGAGGAGGGAGGCAGCAGCTCCAGGCCTGGAC
TGGCCGTAGGAGGCAGCTTCGGACCAGGAACATTCGGCAGGACCCCAGGTAGGA
AGGGTCTTGTCAGGGAAGAGCCTCCCAGCAGAGCCGTGCTTTCCTCCTCTGGGGG

CTTCTCTAACCTGAGCAGAAGCAGAAGCTGCTCACACCATGGAACCTTTCTCTGG
ATTAAAGGGACCAGACATCCCATAAAATATAGGAACACATAGTTTTCAGTGCCTTT
GGACTCACAACCATTCAACTTACTGCTGCTCCCCAAGGCTGTACCACTCTGGGGA
CCTCTGACTCCCCAAACGCTCAAGCAGGAAGGATGGACGTCGGGGCTGTTTCCGT
GATGAGCGGGCTGCATTGCTGGAGAGAAATAGTCTGTTCCTCGGACTGACAAGGT
CTGCAAGGGCACCTGAGATC
